# Macro- and Micro-Morphological Properties of the Rotator Cuff Structures in the Chronic Stage of Tendinopathy in Para Swimmers

**DOI:** 10.3390/jcm15062193

**Published:** 2026-03-13

**Authors:** Beata Pożarowszczyk-Kuczko, Oliwia Jabłońska, Bartłomiej Bogdański, Zofia Wróblewska, Sebastian Klich

**Affiliations:** 1 Faculty of Physical Education and Sport, Wroclaw University of Health and Sport Sciences; 51-612 Wrocław, Poland; beata.pozarowszczyk-kuczko@awf.wroc.pl (B.P.-K.); oliwia.jablonska@awf.wroc.pl (O.J.); zofia.wroblewska@awf.wroc.pl (Z.W.); 2 Faculty of Physical Culture, Gdansk University of Physical Education and Sport, 80-336 Gdańsk, Poland; riolan@vp.pl

**Keywords:** thickness, peak spatial frequency radius, shoulder, overhead sport, overloading

## Abstract

**Background/Objectives**: This study aimed to characterize macro- and micro-morphological properties of the supraspinatus tendon (SST) in para swimmers during the chronic stage of rotator cuff tendinopathy, integrating ultrasound assessments of tendon thickness, peak spatial frequency radius (PSFR) for collagen organization, acromiohumeral distance (AHD), and occupation ratio to evaluate subacromial impingement risk. **Methods**: In a cross-sectional design, 43 elite para swimmers (aged 18–30 years, S7–S10 classes with lower extremity impairments) from Para Swimming Team Poland were divided into rotator cuff tendinopathy (RC; n = 22) and asymptomatic control (CON; n = 21) groups. Measurements on the dominant shoulder utilized B-mode ultrasound (Alpinion X-CUBE 90) to assess SST thickness at 5, 10, and 15 mm proximal to the greater tuberosity, PSFR via MATLAB-analyzed spatial frequency spectra, AHD, and occupation ratio. Two-way and one-way ANOVAs assessed group and measurement effects (*p* < 0.05); Pearson correlations examined the relationships between thickness and PSFR. **Results**: Para swimmers with tendinopathy exhibited greater SST thickness across sites (*p* < 0.001, η^2^ = 0.63), higher PSFR at all intervals (*p* ≤ 0.009, η^2^ = 0.53) peaking at 10 mm, wider AHD (*p* = 0.002, η^2^ = 0.21), and lower occupation ratio (*p* < 0.001, η^2^ = 0.44) versus controls. Strong positive correlations linked thickness and PSFR proximally (r = 0.75–0.79, *p* < 0.001). **Conclusions**: Chronic tendinopathy in para swimmers manifests as thickened SST with collagen disarray, altered subacromial space, and impingement risk, distinguishing pathological from healthy tendons. Integrated ultrasound metrics aid diagnosis and inform interventions for overhead athletes with locomotor disorders.

## 1. Introduction

Para swimming is one of the most popular and trained Paralympic sports, which includes different sport classes for physical, visual, and intellectual impairments. Physical impairments are classified into ten sport classes, i.e., S1–S10, where lower classes indicate greater disability [1]. High functional classes (S7–S10) include moderate impairments, such as single upper/lower extremity amputations, dysmelia, or clubfoot [2]. An epidemiological study showed the risk and amount of shoulder injuries and pain among para swimmers [3]. At the Tokyo 2020 Paralympic Games, shoulder injuries were documented as one of the most common injuries among para swimmers, accounting for 21.4% of all reported injuries.

Rotator cuff tendinopathy is described as one of the most often diagnosed cases of shoulder pain and dysfunction [4]. The term “tendinopathy” may indicate a repetitive stress condition associated with increased pain sensations. Morphologically, it might be related to collagen fiber disorganization due to thickening with a simultaneous decrease in functionality [5]. It may affect a huge number of people, including physical workers, sedentary persons, and athletes, especially in overhead sports [6]. Despite its clinical significance, the intrinsic and extrinsic mechanisms underlying the chronic stage of the disease remain incompletely understood.

The chronic stage of tendinopathy leads to disorganization of collagen fibers replaced by type III collagen, increased vascularity with neovascularization, and mucoid degeneration of the extracellular matrix. These intrinsic factors may cause reduced tendon strength and increased pain. These histopathological changes include hypercellularity, focal necrosis, and irregular fiber crimping, contributing to mechanical instability under load [7].

Macro-morphological properties, such as tendon thickness, may describe structural alterations in the tendon due to overuse, overloading, and damage caused by injury. Previous studies have shown alterations in tendon morphology, i.e., thickening in patients [8,9,10] and swimmers [2,11] with rotator cuff tendinopathy. Thickening of the supraspinatus tendon (SST) is related to greater water content, neovascularization, and increased inflammatory process [6,7]. For instance, Klich et al. (2019) showed that swimmers with upper extremity disorders demonstrated thicker SST compared to able-bodied swimmers and those with lower extremity disorders, along with a greater SST occupation ratio of the subacromial space [2].

Micro-morphology of the tendon can be investigated using ultrasound imaging with B-mode. Ultrasound may visualize the banded speckle pattern, which can be described and analyzed by peak spatial frequency radius (PSFR) [12]. The distribution of collagen fibers is arranged in a parallel and orderly array. However, tendinopathy may disrupt this arrangement, leading to altered collagen fiber distribution and a loss of the normal speckle pattern regularity [13].

Despite the recognition of these multiscale changes, comprehensive analyses that integrate both macro-morphological and micro-morphological characteristics of rotator cuff structures, specifically in the chronic stage of tendinopathy, remain limited. In particular, the current literature is limited according to professional overhead athletes, especially disabled swimmers. To date, only one study has investigated SST characteristics in disabled swimmers in high functional classes with upper extremity disorders, revealing a potential risk for subacromial impingement and tendinopathy due to biomechanical abnormalities [2]. Moreover, one study has evaluated tendon micro-morphological differences between individuals with rotator cuff tendinopathy and healthy controls for the first time, highlighting the current gap in understanding whether micro-morphological changes in SST serve as a reliable marker for distinguishing diseased from healthy tendon tissue [12]. A deeper understanding of such changes is essential to elucidate the pathophysiological processes that drive tendon degeneration, and may support the development of more targeted diagnostic markers and therapeutic strategies.

Therefore, this study aimed to systematically characterize the macro- and micro-morphological properties of rotator cuff structures, particularly the supraspinatus tendon, in para swimmers during the chronic stage of tendinopathy. This integrated analysis will combine ultrasound-based assessments of tendon thickness and occupation ratio with PSFR quantification of collagen fiber organization to elucidate structural remodeling and its association with subacromial impingement risk in this population.

## 2. Materials and Methods

### 2.1. Study Design

This study presents a cross-sectional design with a single measure, where (1) macro- and (2) micro-morphological properties of the SST and subacromial space were investigated. Participants were asked to avoid strength training and high-intensity and volume swimming training for at least 48 h before data collection. All measurements were performed on the dominant side of the upper extremity, made by the same skilled examiner with over 10 years of ultrasound imaging expertise to ensure measurement consistency and reliability. This design was selected for efficient group comparisons and identification of morphological differences associated with tendinopathy. All participants read and signed an informed consent form approved by the Senate Research Ethics Committee (project identification code: 26/2016 approval. This study was conducted according to the Declaration of Helsinki.

### 2.2. Participants

Forty-three elite junior and senior para swimmers were recruited from Para Swimming Team Poland, aged 18–30 years. Table 1 summarizes participant characteristics, including demographic details and training profiles. Participants reported training frequency (9 sessions/week) and approximate volume (58 km/week, 18 h/week) as descriptive characteristics. Subjects were divided into two groups, i.e., para swimmers with RC tendinopathy (RC; n = 22) and asymptomatic matched control para swimmers (CON; n = 21). All swimmers were right-handed. The subjects participated in the European Para Youth Games and/or World Para Swimming Championships. The inclusion criteria for the RC tendinopathy group, i.e., (1) low score in the Shoulder Service Questionnaire (<40 points) and (2) diagnosed tendinopathy via ultrasound imaging. Overall, inclusion criteria for both groups were: (1) classification in high medical class from S7 to S10, including lower extremity impairments, such as severe bilateral limb shortening or significant hip/knee joint ankylosis (S7); moderate leg length discrepancies, severe clubfoot (talipes equinovarus) with contractures, or hip dysplasia (S8); unilateral shortening with moderate restrictions or rigid flatfoot (S9); and mild discrepancies (10–20 cm), mild bilateral clubfoot, or congenital short femur/tibia (S10); (2) training experience (<8 years). The exclusion criteria included: (1) current or previous thigh and knee injury or pain symptoms; (2) prior history of surgery in the lower extremity. According to the medical classification, the S7 class includes swimmers scoring between 191 and 215 points, characterized by paralysis of one arm or congenital upper limb defects. The S8 class encompasses swimmers with scores from 216 to 240, typically with a single upper extremity amputation above the elbow or dysmelia. Swimmers classified as S9 and S10, scoring between 241 and 285 points, have lower limb impairments such as joint restrictions or amputations (S9), with S10 representing those with minimal physical impairments [14].

### 2.3. Experimental Procedure

#### 2.3.1. Ultrasound Imaging Specificity

The SST and subacromial space images were performed using ultrasonography (Alpinion X-CUBE 90, Opinion, Seoul, Republic of Korea) with a linear array transducer (3.0 to 19.0 MHz; 60 mm; SL3-19X; X+ Crystal Signature™; Alpinion; Seoul, Republic of Korea) in grey-scale B-mode. Ultrasound imaging included macro- (thickness [mm], subacromial space defined as the acromiohumeral distance (AHD, [mm]), and occupational ratio [%]) and micro-morphological (peak spatial frequency radius (PSFR [mm^−1^]) properties.

For optimal ultrasound imaging of the SST and subacromial space, the examiner selected a high-frequency linear transducer (18 MHz). However, for speckle tracking of the micro-morphology (i.e., PSFR) of the tendon, a lower frequency of the transducer was used (to 8 MHz). Next, the ultrasound machine was preset for musculoskeletal imaging with opacity set at 100%, depth at 2–4 cm to frame the tendon insertion with an extra 1 cm of the humeral head, the focus placed at 1–2 cm depth for sharp resolution, and overall gain adjusted to 50–70% with TGC curved upward near the superficial tendon to ensure uniform brightness without saturation.

Once the ultrasound machine was set, the examiner informed the participant about the goal of the measurements, positioning, and further processing of the images. After each data collection, the examiner saved each image on the ultrasound machine and then to an external disk.

#### 2.3.2. Macro-Morphology of the Rotator Cuff

##### Supraspinatus Tendon (SST) Thickness

The participant was asked to seat on a chair, with arms placed at the side of the body in a neutral position, thumbs pointing forward, with their feet flat on the floor, and knees and hips flexed at approximately 90° as described in previous studies [2,15]. During the examination, the transducer was moved anteriorly and laterally until the most distal end of the SST with the hyperechoic greater tuberosity was defined. In the longitudinal view, the SST appears as a beak-shaped structure with a fibrillar pattern and regular margins. Finally, the transducer was gently moved side to side with slight pressure toward the patient’s body. The SST was defined on the anterior aspect of the shoulder, just off the acromion, oriented approximately 45° between the sagittal and frontal planes [2,15].

**Figure 1:**
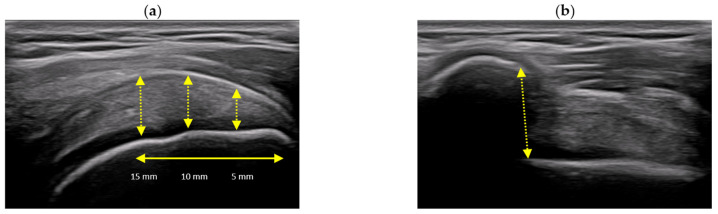
Ultrasound B-mode imaging, including (**a**) supraspinatus tendon in longitudinal view; thickness measure procedure at 5 mm, 10 mm, and 15 mm distances from the most hyperechogenic point at the greater tuberosity; and (**b**) subacromial space with acromiohumeral distance measure.

The measurement procedure included three segmental thickness measurements at 5, 10, and 15 mm proximal to the attachment on the greater tuberosity. The tendon boundaries were identified as the most hyperechoic area above the anechoic articular cartilage of the humeral head and the hyperechoic upper edge of the tendon anterior to the anechoic subacromial–subdeltoid bursa (Figure 1) [2].

##### Subacromial Space

The subacromial space images were captured in the same position as for the SST, except that the arm was placed on the thigh in a resting position. Next, the ultrasound transducer was placed on the anterior acromial margin to identify the anterolateral aspect. The AHD was measured as a linear distance between the inferior aspect of the acromion and the superior aspect of the humeral head (Figure 1) [2,15].

##### Occupational Ratio

The occupation ratio was calculated as the mean tendon thickness expressed as a percentage of the mean AHD [9]. The equations present the exact calculation for the occupation ratio.

(1) $$Occupational\;ratio\;\left[\%\right]=\frac{SST\;thickness}{AHD}\times 100\%$$

#### 2.3.3. Micro-Morphology of the Rotator Cuff

**Figure 2:**
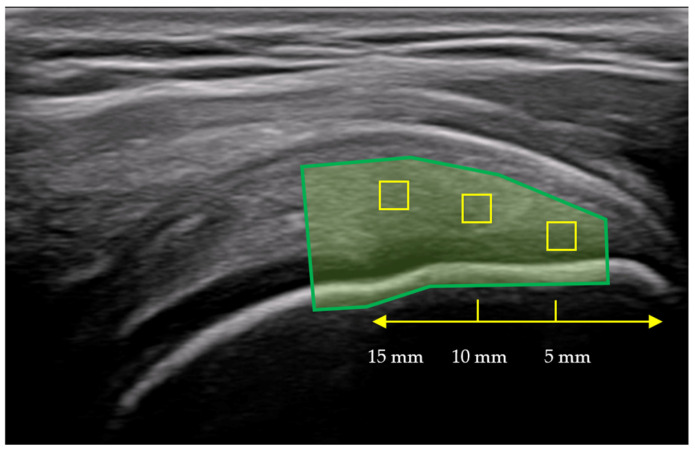
Ultrasound B-mode of the supraspinatus tendon with reference points for the measurement of peak spatial frequency radius (PSFR) at 5 mm, 10 mm, and 15 mm distances from the most hyperechogenic point at the greater tuberosity.

**Figure 3:**
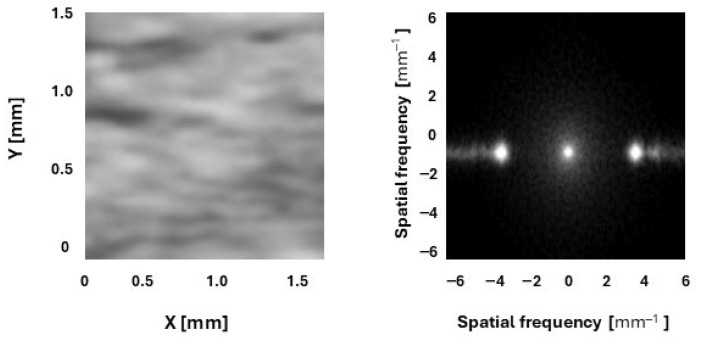
Example images demonstrating kernel and spatial frequency spectrum.

A B-mode ultrasound image of the SST was stored as a Digital Imaging and Communications in Medicine (DICOM) file. It is a typical image format used in medical imaging, such as ultrasound, magnetic resonance, and X-ray. Next, the DICOM file was opened in MicroDicom DICOM Viewer (MicroDicom Ltd., Sofia, Bulgaria), where the best view from a 6 s image loop was captured and saved as a JPG file for PSFR analysis. The analysis was conducted in two stages: First, a region of interest (ROI) was selected on the B-mode image to define the entire tendon, from the humeral head to the bursal layer of the subdeltoid bursa. Next, the images were analyzed using a custom MATLAB 2025b algorithm (MathWorks, Natick, MA, USA). Spatial Frequency Analysis (SFA) using SpaFTA 2.6 in MATLAB enables quantitative assessment of tendon micro-morphology by evaluating PSFR. For each image, three kernels were marked and analyzed using a 2D Fast Fourier Transform. The kernels were placed in three 5 mm intervals at the most echogenic area of the tendon—specifically at 5 mm, 10 mm, and 15 mm from the greater tuberosity. The kernel at 5 mm was a square measuring 24 × 24 pixels (1.05 × 1.05 mm), at 10 mm it was 32 × 32 pixels (1.4 × 1.4 mm), and at 15 mm it was 38 × 38 pixels (1.66 × 1.66 mm) (Figure 2). The kernel was expanded to 128 × 128 samples via zero padding. A two-dimensional high-pass filter with a 3 dB cutoff frequency of approximately 1.0 mm^−1^ was then applied to reduce low spatial frequency artifacts. The resulting PSFR (mm^−1^) values from the three images were averaged and used for further analysis (Figure 3). Higher PSFR values indicate greater collagen fiber organization [12,16].

### 2.4. Statistical Analysis

The SPSS 26 statistical software (SPSS Inc., Chicago, IL, USA) was used for data analysis. Mean values ± standard deviations (SD) are reported. The normality of the data distribution was checked using the Shapiro–Wilk tests, while homogeneity of variance was analyzed by Levene’s test. The analyzed data were normally distributed for all parameters, while the variances for all parameters were equal. Tendon thickness and PSFR were compared between groups (i.e., RC group, control group) using a one-way analysis of variance (ANOVA). The effect size was estimated using partial eta squared (η^2^), classified as small (0.2–0.49), medium (0.5–0.79), or large (0.8 ≤ η^2^). We evaluated the relationships between tendon morphology and collagen organization by calculating Pearson correlation coefficients (r) between longitudinal tendon thickness and PSFR for both involved and uninvolved sides. For all statistical analyses, including mixed ANOVA tests and Pearson correlation coefficients, a *p*-value < 0.05 was considered significant.

## 3. Results

### 3.1. Macro-Morphology of the Rotator Cuff

#### 3.1.1. Supraspinatus Tendon (SST) Thickness

**Figure 4:**
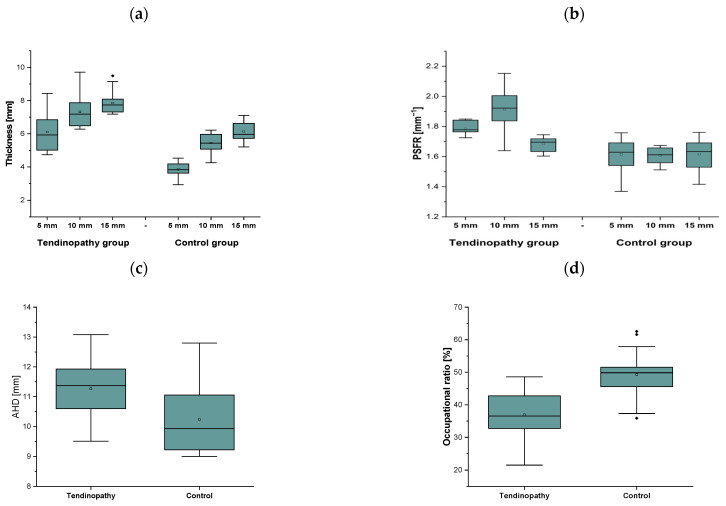
Mean ± SD of the supraspinatus macro- and micro-morphological properties, including (**a**) supraspinatus tendon thickness [mm]; (**b**) peak spatial frequency radius (PSFR) [mm^−1^]; (**c**) acromiohumeral distance (AHD) [mm]; and (**d**) occupational ratio [%] in para swimmers with chronic rotator cuff tendinopathy (Tendinopathy) and a control group (Control).

Figure 4a demonstrates the mean ± SD of SST thickness at three different measurement intervals (i.e., at 5, 10, and 15 mm) in para swimmers with diagnosed tendinopathy and the control group. The two-way ANOVA only revealed a significant main effect of *Group* (F_1,126_ = 212.4; *p* ≤ 0.001; η^2^ = 0.63) and *Measure* (F_2,126_ = 79.4; *p* ≤ 0.001; η^2^ = 0.56). Overall, SST thickness was larger in para swimmers with rotator cuff tendinopathy compared to the control group (*p* < 0.001), as well as at 15 mm distantly from the attachment to the greater tuberosity compared to the interval measures at 5 mm and 10 mm (*p* < 0.001 and *p* = 0.001, respectively).

#### 3.1.2. Subacromial Space and Occupational Ratio

The one-way ANOVA only revealed a significant main effect of *Group* (F_1,42_ = 11.0; *p* = 0.002; η^2^ = 0.21) for AHD. Overall, AHD was wider in para swimmers with rotator cuff tendinopathy compared to the control group (*p* = 0.002) (Figure 4c). For the occupational ratio, the one-way ANOVA only revealed a significant main effect of *Group* (F_1,42_ = 32.8; *p* ≤ 0.001; η^2^ = 0.44). Overall, the occupational ratio was greater in the control group compared to the tendinopathy group (*p* < 0.001) (Figure 4d).

### 3.2. Micro-Morphology of the Rotator Cuff

#### Peak Spatial Frequency Radius (PSFR)

The two-way ANOVA revealed a significant main effect of *Group* (F_1,126_ = 142.6; *p* ≤ 0.001; η^2^ = 0.53) and *Measure* (F_2,126_ = 17.2; *p* ≤ 0.001; η^2^ = 0.21) with an interaction effect between *Group* and *Measure* (F_2,126_ = 18.9; *p* ≤ 0.001; η^2^ = 0.24) for PSFR. The post hoc analysis showed greater PSFR in para swimmers with tendinopathy at 5 mm, 10 mm, and 15 mm compared to the control group (*p* < 0.001, *p* < 0.001, and *p* = 0.009). Moreover, PSFR was larger at 10 mm compared to 5 mm and 15 mm (*p* < 0.001 for both) and at 5 mm compared to 15 mm (*p* = 0.001) in the tendinopathy group (Figure 4b).

The interaction between SST thickness and groups was not statistically significant (β = 0.0055, *p* = 0.746). This finding indicates that the association between SST thickness and PSFR does not differ between the tendinopathy and control groups. Overall, tendon thickness shows a similar relationship with PSFR across all measurement intervals in both groups.

**Figure 5:**
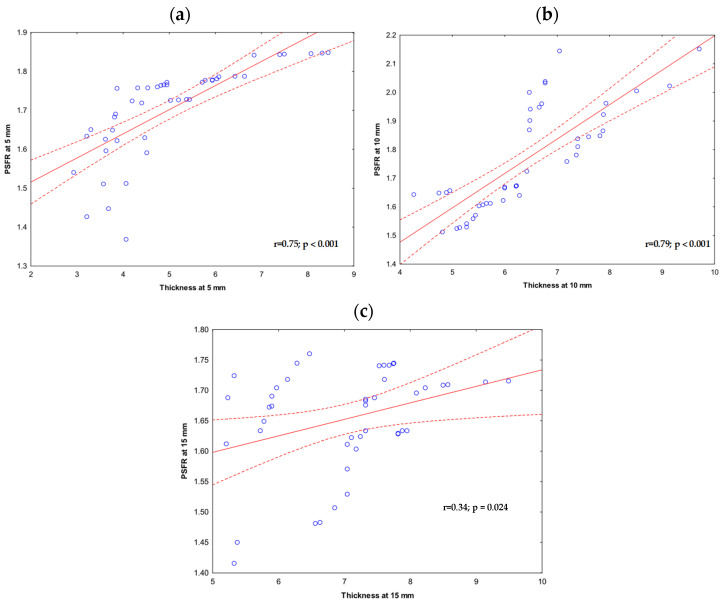
Ultrasound between supraspinatus tendon thickness and peak spatial frequency radius (PSFR) at (**a**) 5 mm; (**b**) 10 mm; and (**c**) 15 mm distantly from the most hyperechogenic point at the greater tuberosity.

There was a strong positive correlation between thickness and PSFR at 5 mm (r = 0.75, *p* < 0.001) and 10 mm (r = 0.79, *p* < 0.001). At 15 mm, the correlation was low, albeit significant (r = 0.34, *p* = 0.024) (Figure 5a–c).

## 4. Discussion

The SST was structurally characterized by macro- and micro-morphological properties in the rotator cuff. The ultrasound imaging analysis showed signs of chronic tendinopathy in para swimmers. The study involves novel evidence of integrated ultrasound assessments of tendon thickness, PSFR for collagen organization, AHD, and occupation ratio in elite para swimmers, as potential risk factors of rotator cuff dysfunction. Prior studies reported epidemiological data indicating an increased incidence of shoulder injuries during the Tokyo 2020 Paralympic Games. In summary, our results showed a thicker SST with greater collagen organization (higher PSFR), a wider subacromial space, and a lower occupation ratio. These chronic tendinopathy outcomes suggest the development of subacromial impingement. Finally, the strong positive correlations between thickness and PSFR in the proximal segment of the tendon may suggest thickening as a result of tendon damage rather than adaptive hypertrophy.

Para swimmers with chronic rotator cuff tendinopathy reported greater SST thickness along with a wider subacromial space. These observations may also suggest chronic subacromial impingement, because the overall occupation ratio is lower compared to the control group. Swimming, as a repetitive overhead sport, may increase muscle loading and generate a higher degree of stress in athletes [1]. Physiologically, a thicker tendon may be related to increased vascularity, inflammatory process, and water accumulation [9,17]. Tsui et al. (2007) reported greater vascularity in rotator cuff tendinopathy, which was related to reduced AHD [18]. A previous study by Klich et al. (2019) also showed subacromial impingement symptoms, such as a lower occupational ratio in para swimmers compared to able-bodied swimmers [2]. Chronic tendinopathy has been described in the continuum model of tendon pathology [19]. The model classified pathology into two groups, i.e., reactive and degenerative tendinopathy. Reactive tendinopathy might be associated with greater thickness and hypoechoic regions observed on ultrasound imaging. On the other hand, degenerative tendinopathy is frequently linked to tendon thinning and tissue deterioration [19,20,21]. This study did not investigate the morphological properties of these two types of tendinopathy. Most probably, two types of tendinopathy may represent opposite adaptations in the tendon’s morphology; therefore, distinguishing between them is essential for accurate diagnosis.

This study demonstrated greater PSFR at multiple segments compared to the control group, indicating more collagen disorganization [22]. The PSFR is differentiated along the tendon length, with the highest PSFR observed at 10 mm in para swimmers with tendinopathy. There was a strong positive correlation between tendon thickness and PSFR at the proximal end (10 mm), suggesting that thicker tendons had greater structural alterations. Histologically, SST is built over five layers with a different structure and collagen organization [23]. Moreover, the bursal- and joint-side layer is characterized by a completely different content of muscle and tendon fibers [24]. According to Nakajima et al. (1994), the middle portion of the SST (approx. 12 mm from the greatest tuberosity) may be exposed to damage and tears [24]. It is commonly called the critical zone [24]. Our study may confirm these observations, especially by higher PSFR values in the middle portion (at 10 mm). Technically, PSFR uses anisotropic speckle patterns obtained during ultrasound imaging [16]. This speckle analysis may define structural alterations and damage of a tendon in tendinopathy. Increased PSFR may suggest early degenerative alterations with proliferation of type III collagen and mucoid changes [25]. Unlike other studies, our results indicate greater PSFR in para swimmers with tendinopathy compared to the control group. Pozzi et al. (2017) reported no differences between tendinopathy and a control group [22]. Moreover, the magnitude of PSFR was also higher in our study compared to previous studies [22]. Finally, proximal thickness and PSFR correlations were reported in para swimmers with tendinopathy, which may suggest tendon thickening as a result of damage from training-induced adaptive hypertrophy.

Observed differences in macro- and micro-morphological properties of the rotator cuff may indicate higher loading on the tendon during repeated overhead movements during swimming and potential adaptation of the tendon to disability in Paralympic athletes. Although we observed significant morphological differences between groups, the cross-sectional design prevents causal inference. Observations from this study should be interpreted with caution because they are the result of uncorrected univariate analyses conducted on a very specific elite cohort from a single national team. Despite observing significant differences in morphological properties between groups, these univariate comparisons lack multivariable adjustment and should be interpreted cautiously due to potential confounding.

This study has some potential limitations. Since this research employed a cross-sectional design with single-timepoint measurements, causal inferences cannot be made. The observed differences may precede, result from, or reflect bidirectional associations with the development of tendinopathy. The lack of biomechanical analysis and functional assessment is another limitation that prevents consideration of the main confounding factors of tendinopathy. Future studies could adopt a longitudinal design to establish a timeline of pathological changes in the tendon, while future studies with larger samples should employ regression models adjusting for age, training experience, disability characteristics, and workload to confirm these group differences. Moreover, future studies should focus on differences between reactive and degenerative tendinopathy subtypes.

## 5. Conclusions

Both thickness and PSFR are useful measures of outcomes for evaluating rotator cuff tendinopathy and signs of chronic degeneration of SST. This study revealed greater SST thickness and higher PSFR in para swimmers with chronic rotator cuff tendinopathy. Morphological alterations in the rotator cuff may be associated with subacromial impingement mechanisms, as a result of a lower occupation ratio in para swimmers with supraspinatus tendinopathy. Repetitive overhead arm movements during swimming increase mechanical stress on the subacromial space, potentially leading to progressive tendon compression. Integrated ultrasound assessments of macro- and micro-morphological properties provide valuable insights for diagnosis and targeted interventions in overhead athletes with locomotor disorders.

## Figures and Tables

**Table 1:** Mean ± SD of the participant characteristics.

Variables	RC Group	CON Group
Age (year)	25 ± 2	22 ± 1
Sex Body height (m)	♂: 11; ♀: 11 1.70 ± 3.1	♂: 10; ♀: 11 1.72 ± 3.5
Body mass (kg)	70.47 ± 4.2	68.30 ± 3.7
Body mass index (kg/m^2^) Rotator cuff tendinopathy: Right Left	24.0 ± 0.3 n = 22 n = 0	23.0 ± 0.5 N/A N/A
Training experience (years)	11 ± 3	10 ± 1

RC group—para swimmers with RC tendinopathy, CON group—asymptomatic matched control para swimmers, N/A—not applicable.

## Data Availability

The data supporting the findings of this study are available from the authors upon request.

## Abbreviations

SST	Supraspinatus tendon
PSFR	Peak spatial frequency radius
ROI	Region of interest
AHD	Acromiohumeral distance
RC	Rotator cuff
CON	Control group
ANOVA	Analysis of variance
